# Aging and the impact of global DNA methylation, telomere shortening, and total oxidative status on sarcopenia and frailty syndrome

**DOI:** 10.1186/s12979-023-00384-2

**Published:** 2023-11-14

**Authors:** Tomasz Kmiołek, Gabriela Filipowicz, Diana Bogucka, Anna Wajda, Adam Ejma-Multański, Barbara Stypińska, Ewa Modzelewska, Yana Kaliberda, Marcin Radkowski, Tomasz Targowski, Julia Wrona, Agnieszka Paradowska-Gorycka

**Affiliations:** 1 Department of Molecular Biology, National Institute of Geriatrics, Rheumatology and Rehabilitation, 02-637 Warsawm, Poland; 2 Department of Pathophysiology and Immunology, National Institute of Geriatrics, Rheumatology and Rehabilitation, 02-637 Warsaw, Poland; 3 Collegium Medicum University of Jan Kochanowski, 25-317 Kielce, Poland

**Keywords:** Aging, Telomere length, DNA methylation, Total oxidative stress, Sarcopenia, Frailty syndrome

## Abstract

Aging is a biological event that influences many organs and systems. Both sarcopenia and frailty syndrome refer to geriatric conditions with overlapping phenotypes. Many mechanisms are involved in the aging process such as DNA methylation telomeres which are susceptible to oxidative stress, and inflammations which result in telomere shortening, leading to chromosomal instability. The study aimed to determine the associations between these processes, frailty and sarcopenia syndrome. Global DNA methylation was analyzed using the ELISA method. Telomere length was analyzed using qPCR. Total oxidative status (TOS) was analyzed using a colorimetric method. The present study revealed that the main factor affecting methylation, telomeres length and level of total oxidant stress was age.

## Introduction

Aging is a complex biological event that has an influence on many organs and systems' functions. Aging is a major factor in geriatric diseases [1]. Inflammation, stress adaptation, and alteration of metabolic pathways are the ground of aging and age-related diseases. Additionally, exceptional changes occur in the immune system called “immunosenescence” which leads to inflammaging [2]. This relatively new term defines the lower type of chronic inflammatory status which is distinctive to the aging process [3]. Moreover, a lot of different age-related entities like atherosclerosis, cardiovascular diseases, cognitive decline, type 2 diabetes, sarcopenia, and osteoporosis, share a common underlying inflammatory pathogenesis [4, 5].

Epigenetic mechanisms, especially DNA methylation, can influence changes in the molecular mechanisms of aging. DNA methylation is catalyzed by DNMT (DNA methyltransferase) and a methyl group is attached to the 5’ carbon position, in particular, of cytosine (5mC; 5-methylcytosine) in the CpG dinucleotides [6]. CpG motif methylation occurs in a cell and tissue-specific manner, which has a decisive impact on their differentiation and development. The level of gene expression correlates with the amount of methylated DNA in the promoter sequence [7]. The hypermethylation of the promoter leads to gene inactivation, while hypomethylation of the gene promoter can contribute to the activation of gene expression [8, 9]. The role of DNA methylation in aging and the development of other diseases, such as cancers, has been widely investigated, but a clear understanding of its role in muscle aging is still poorly known [10, 11].

Another crucial role in aging and genome stability is played by telomeres. Telomeres are dynamic nucleoprotein structures which protect the ends of chromosomes from degradation and activation of the DNA damage response. Therefore, they also have the potential to be used as biomarkers for many age-related diseases [12]. Telomeres are vulnerable to oxidative stress, as well as to inflammation [13]. This vulnerability causes shortening of the telomere which consequently leads to chromosomal instability. To prevent this process, telomeres are elongated by telomerase, which is a cellular reverse transcriptase [14]. Telomerase is used for the treatment of age-related diseases and telomeropathies [15]. Also, anti-inflammatory agents and antioxidants can find application to slow telomere length loss [16].

Sarcopenia and frailty are age-related syndromes. Both entities are geriatric terms with overlapping phenotypes and are characterized by reduced quality of life, physical disability and consequently lead to death. In the elderly population, sarcopenia occurs in about 10% of people, and frailty syndrome is observed in 15% of the population. However, with chronic diseases and multi-morbidity, the prevalence increases to 20% and 60%, respectively [17]. Sarcopenia is a degenerative and progressive type of skeletal muscle atrophy that usually precedes frailty [6, 7]. Frailty is a syndrome characterized by increasing vulnerability to stressors, resulting in impaired regulation in multiple organs, which causes reduced autonomy, exhaustion, muscle weakness and unintended weight loss, and consequently loss of independence for the elderly [6, 8, 11, 18]. Sarcopenia has been identified as a genetic expression of apoptosis, muscular autophagy factors and vitamin D receptors, chronic inflammation, telomere shortening, and oxidative stress [19, 20]. Frailty is correlated mainly with inflammation pathways, nonspecific immunological alteration related to immunosenescence, and oxidative stress [21]. Nevertheless, both syndromes have common features. Moreover, they have similar therapeutic approaches which are based on physical activities and proper nutrition [22].

Therefore the main aim of the present study was to determine the association between shortening of telomeres, global methylation and total oxidant status in patients with frailty and sarcopenia syndrome. Understanding the molecular mechanisms leading to the development of sarcopenia, frailty and the difference between these two syndromes will allow for better diagnosis and also allow for more accurate treatment.

## Patients and methods

### Patients

The study was conducted in the National Institute of Geriatrics, Rheumatology, and Rehabilitation, Warsaw Poland. Blood samples were collected from immunocompetent patients with sarcopenia (*n* = 12), frailty syndrome (*n* = 30) and older than 60 years patients without the above mentioned entities (*n* = 27). People with malignant solid tumors, active inflammatory or autoimmune diseases, proliferative diseases of the hematopoietic system or taking immunosuppressive drugs were excluded from the study. Additionally, whole blood samples were collected from two groups of healthy subjects: between 25–30 years old and above 50 years old. This study met all criteria contained in the Declaration of Helsinki and was approved by the Ethics Committee of the National Institute of Geriatrics, Rheumatology, and Rehabilitation, Warsaw, Poland (approval protocol number KBT-4/2/2018). All participants gave their written informed consent before enrollment. All patients with sarcopenia fulfilled the European Working Group on Sarcopenia in Older People criteria from 2010, patients with frailty were qualified after meeting three out of five phenotypical criteria defined by Fried et al. [23]. Control group consisted of healthy volunteers, matched, gender and ethnicity of studied patients. Patients selected for this study were evaluated based on physical examination and laboratory tests. Age, gender, disease duration, DEXA (Dual-energy x-ray absorptiometry) measures BMC (bone mineral content), CGE (Comprehensive geriatric evaluation). The presence of diabetes, osteopenia, osteoporosis, and nicotinism. Additionally results of VES-13 (Vulnerable elder scale-13), FI-CGA (Frailty index-comprehensive geriatric assessment), ADL (Activities of daily living), IADL (Instrumental activities of daily living), Barthel scale, Tinetti POMA (Performance oriented mobility assessment) test, and TUG (Time up and go) test. Vitamins were measured in the National Institute of Geriatrics, Rheumatology and Rehabilitation, through a central clinical laboratory using the chemiluminescence method.

### DNA isolation

DNA was extracted from whole blood using an A&A Biotech Blood Mini kit (A&A Biotechnology, Poland) according to the manufacturer’s standard protocol. The quantity and quality of samples were measured with DeNovix (DeNovix Inc., Wilington, USA). The purity of the DNA samples was calculated to a 260/280 nm OD ratio with expected values between 1.8 and 2.0. DNA was stored at -80 °C until required for further analysis.

### Global DNA methylation assessment

Global DNA methylation levels were analyzed in 80 ng genomic DNA using the ELISA-based commercial kit (MethylFlash Global DNA methylation (5-mC) ELISA Easy Kit (Colorimetric), EpiGentek Group Inc.) following the manufacturer’s instruction. The percentage of methylated DNA (5-MC%) in the total DNA sample was calculated using a standard curve generated by the absorbance values of six concentration points (0.1–5% methylated DNA) according to the manufacturer’s instructions. Results were read at 450 nm using a microplate reader Tecan Infinite F PLEX (Tecan Group Ltd., Switzerland). Analysis was performed using MAGELLAN PRO V7.4 STD.2PC software.

### Telomere length

For telomere length analysis Relative Human Telomere Length Quantification qPCR Assay Kit (ScienCell USA) and QuantStudio 5 detection system (Life Technologies, Carlsbad, CA, USA) were used. For each DNA sample two qPCR reactions were prepared:1) with telomere primer solution (TLR) and 2) single copy reference (SCR) primer solution (which was a reference copy). Reaction consisted of 1 μL of DNA, 2 μL of TLR/SCR primer, 10 μL of 2xGoldNStarrt TaqGreen qPCR master mix, and 7 μL of nuclease-free H_2_O. The plate was placed in a real-time cycler (QuantStudio 5; Applied Biosystem). PCR condition was set following manufactures protocol.

#### Analysis

Analysis of relative telomere length (RTL) was conducted by comparative ∆∆Cq method, according to the manufacturer’s recommendation. As a reference group, geriatric patients without sarcopenia or frailty syndrome were taken.

$$\begin{array}{c}\Delta \mathrm{Cq }\left(\mathrm{TEL}\right)= {\mathrm{Ct}}_{\mathrm{TEL of the individual patient}} - {\mathrm{Ct }}_{\mathrm{TEL mean of the reference group}}\\ \Delta \mathrm{Cq }\left(\mathrm{SCR}\right)= {\mathrm{Ct}}_{\mathrm{SCR of the individual patient}} -{\mathrm{Ct}}_{\mathrm{SCR mean of the reference group}} \\ \begin{array}{c}\Delta \Delta \mathrm{Cq }= \Delta \mathrm{Cq }\left(\mathrm{TEL}\right)-\Delta \mathrm{Cq }(\mathrm{SCR})\\ \mathrm{RTL }= {2}^{- \Delta \Delta \mathrm{Cq}}\end{array}\end{array}$$

### Total oxidant status

Total Oxidant Status was analyzed in patients’ serum using the commercially available Total Oxidant Status (TOS) Colorimetric Assay Kit (Elabscience, China), according to the manufacturer’s protocol. The overall oxidant status of the samples (in µmol H2O2 equiv./L) was assessed by using an eight point standard curve (with concentrations ranging from 0 to 100 µmol/L), as recommended in the manufacturer's manual. The spectrophotometric analysis was performed using a microplate reader Tecan Infinite F PLEX (Tecan Group Ltd., Switzerland) set to 600 nm wavelength.

### Statistical analysis

Normality distribution has been checked by the Shapiro–Wilk test and Levene’s Test was used for Homogeneity of Variance. Comparison between analyzed groups was conducted by Anova and post hoc Tukey test or Kruskall-Wallis with Dunn’s test. Additionally, correlation has been analyzed by Pearson or Spearman test. Statistical significance of relative telomere length in analyzed groups in comparison to the calibrator group was analyzed using Mann–Whitney test. Total oxidant status of the five groups was statistically analyzed using Kruskall – Wallis and Dunn’s multiple comparisons tests. RStudio Version 1.4.1717 © 2009–2021 RStudio, and GraphPad Prism 9.5.1 were used to conduct analysis and present graphs.

## Results

### Patients

Patients in this study were divided into three research groups (Sarcopenia with 12 patients whose age was between 52–95 years old, frailty syndrome with 30 patients whose age was between 64–98, and Geriatrics control with 27 patients whose age was between 61–88), and two control groups (healthy volunteers whose age was between 21–30 median 27 named as 25–30, and healthy volunteers whose age was between 47–74 median 54.5 called as group 50 +). No statistical difference in age was found between patients with frailty syndrome vs sarcopenia vs geriatric patients. All of the above-mentioned groups were significantly older than healthy subjects from the group 50 + . Due to technical reasons, not all patients were included in every experiment. Demographic and clinical characteristics of patients with p-value were summarized in Table 1.

**Table 1:** Demographic and clinical characteristics of patients with sarcopenia and frailty syndrome and geriatric control

	**Sarcopenia (*N* = 12)**	**Frailty syndrome (*N* = 36)**	**Geriatric control (*n* = 25)**	***p*-value**
Age (years, mean ± SD)	77.33 ± 13.75	80.50 ± 8.72	72.56 ± 13.75	0.004*
Gender				0.45***
Women n(%)	6 (50.00%)	25 (69.44%)	17 (68.00%)	
Men n(%)	6 (50.00%)	11 (30.56%)	8 (31.00%)	
Mean ± SD				*p*-value*
ASMM (kg)	5.95 ± 0.90	7.19 ± 0.74	7.47 ± 0.80	< 0.0001
FI-CGA	0.21 ± 0.11	0.36 ± 0.09	0.14 ± 0.06	0.004
BMI (kg/m^2)	23.18 ± 2.97	27.61 ± 6.37	29.86 ± 5.28	0.005
Cholesterol (mg/dl)	194.50 ± 42.75	173.85 ± 41.31	204.24 ± 42.99	0.022
Triglycerides (nmol/l)	105.58 ± 42.91	138.74 ± 66.59	147.04 ± 60.18	0.154
LDL (mg/dL)	100.65 ± 37.64	91.06 ± 30.08	120.50 ± 37.65	0.006
Albumin (g/dl)	4.17 ± 0,38	3.98 ± 0.49	4.10 ± 0.32	0.289
Hemoglobin (g/dl)	12.50 ± 1.60	12.11 ± 1.73	13.29 ± 1.43	0.023
LDH (U/L)	260.52 ± 61.35	252.89 ± 103.61	229.81 ± 41.67	0.443
Creatine (µmol/l)	0.97 ± 0.29	0.88 ± 0.24	0.89 ± 0.27	0.605
Vitamin D3 (ng/ml)	33.75 ± 34.43	36.81 ± 29.63	35.09 ± 34.88	0.850
Vitamin B12 (pg/ml)	455.65 ± 245.43	397.72 ± 187.11	426.20 ± 243.40	0.704
Serum Fe level (mcg/dl)	79.96 ± 10.50	61.16 ± 22.84	84.11 ± 22.97	< 0.0001
Median (IQR)				*P*-value**
TUG (s)	14.50 (11.70, 21.97)	200.00 (14.75, 26.50)	14.00 (13.00, 15.00)	< 0.0001
ESR (mm/h)	11.00 (8.00, 15.50)	22.00 (12.50, 35.25)	10.00 (8.00, 22.00)	0.048
CRP (mg/dl)	5.00 (5.00, 6.00)	7.00 (5.00, 25.50)	5.00 (5.00, 12.00)	0.062
NT-proBNP (pg/ml)	354.80 (277.88, 666,57)	264.30 (163.67, 510.20)	258.60 (164.60, 559.60)	0.308
CK (U/I)	67.00 (55.00, 70.79)	71.00 (45.25, 86.50)	72.00 (56.00, 106.00)	0.382
N (%)				*p*-value***
Osteopenia				0.009
Present	6 (50.00%)	4 (11.11%)	2 (8.00%)	
Not present	6 (50.00%)	32 (88.89)	23 (93.00%)	
NYHA class				0.153
0	7 (58.33%)	27 (75.00%)	23 (92.00%)	
I	0 (0.00%)	1 (2.78%)	0 (0.00%)	
II	3 (25.00%)	6 (16.67%)	1 (4.00%)	
II-III	2 (16.67%)	1 (2.78%)	1 (4.00%)	
III	0 (0.00%)	1 (2.78%)	0 (0.00%)	
Polyarthritis				
Present	4 (33.33%)	18 (50.00%)	13 (52.00%)	0.574
Not present	8 (66.67%)	18 (50.00%)	12 (48.00%)	
Osteoporosis				
Present	3 (25.00%)	9 (25.50%)	4 (16.00%)	0.691
Not present	9 (75.00%)	27 (75.00%)	21 (84.00%)	

*Abbreviations ADL* Activities of daily living, *ASMM* Appendicular skeletal muscle mass, *BMI* Body mass index, *CRP* C-reactive protein, *DEXA* Dual-energy X-ray absorptiometry, *FI-CGA* Frailty index-comprehensive geriatric assessment, *LDL* Low-density lipoprotein, *POMA* Performance-oriented mobility assessment, *TUG* Timed up & go, *VES* Vulnerable elders survey, *in comparison to the group 50 +

^*^One-way analysis of means (not assuming equal variances) or analysis of variance

^**^Kruskal–Wallis rank sum test

^***^Person’s Chi-squared test or Fisher’s Exact Test for Count Data

**Fig. 1:**
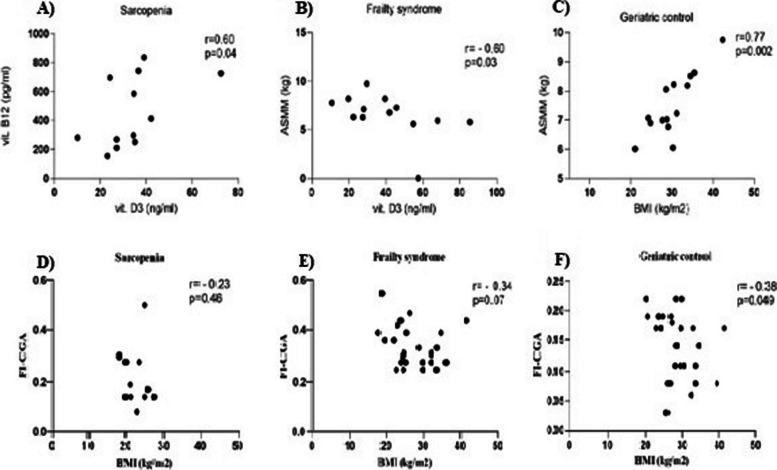
Correlation between level of vitamin B12 and vitamin D3 in patients with **A**) sarcopenia, **B**) ASMM and level of vitamin D3 in patients with frailty syndrome, **C**) between ASMM and BMI in geriatric control, between level of FI-CGA and BMI in patients with **D**) sarcopenia, **E**) frailty syndrome and **F**) geriatric control

We found a statistically significant correlation between clinical characteristics in different patient groups. A positive correlation between levels of vitamin B12 and vitamin D3 (*r* = 0.60, *p* = 0.04) was noted in sarcopenia patients. Patients with frailty syndrome were characterized with a negative correlation between ASMM (Appendicular skeletal muscle mass) and level of vitamin D3 (*r* = -0.60, *p* = 0.03), and correlation analysis between the level of FI-CGA and BMI showed a negative correlation in geriatric control (*r* = -0.38, *p* = 0.049, Fig. 1).

Additionally we analyzed the correlation between ASMM and the percentage of methylated DNA, FI-CGA and BMI, ASMM and Tug, and between vitamin B12 with a percentage of methylated DNA.

**Fig. 2:**
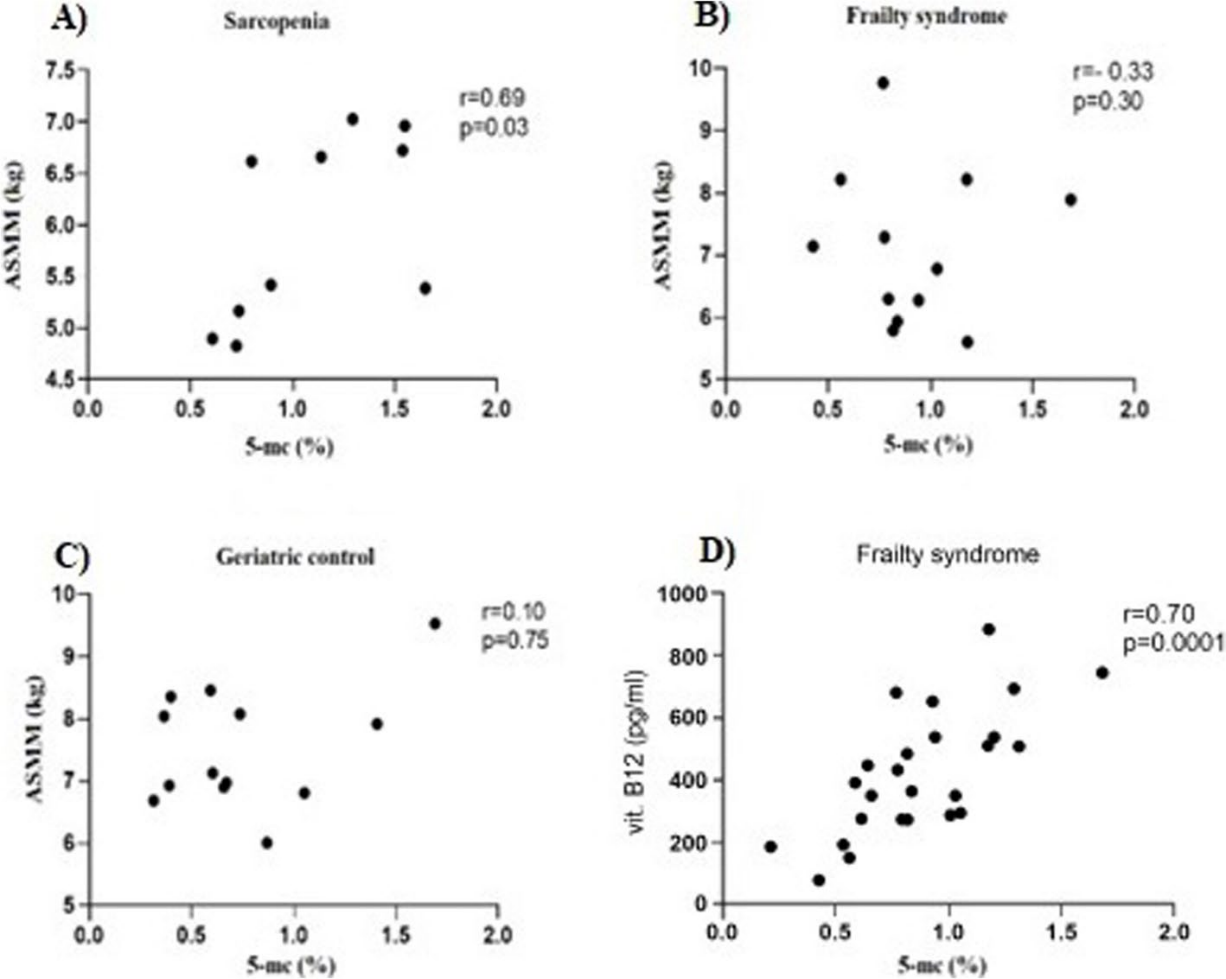
Correlation between level of ASMM and percentage of methylated DNA (5-mc) in patients with **A**) sarcopenia, **B**) frailty syndrome, **C**) geriatric control, and correlation between level of vitamin B12 and 5-mc in patients with frailty syndrome

In the analysis of the correlation between ASMM and 5-mc, we were able to find a statistically significant positive correlation in patients with sarcopenia (*r* = 0.69, *p* = 0.03, Fig. 2). We did not find any statistically significant correlation between ASMM and Tug in any group of patients participating in the study. However, we found a positive correlation between the level of vitamin B12 and the percentage of methylated DNA in patients with frailty syndrome (*r* = 0.70, *p* = 0.0001, Fig. 2).

### Global methylation

**Fig. 3:**
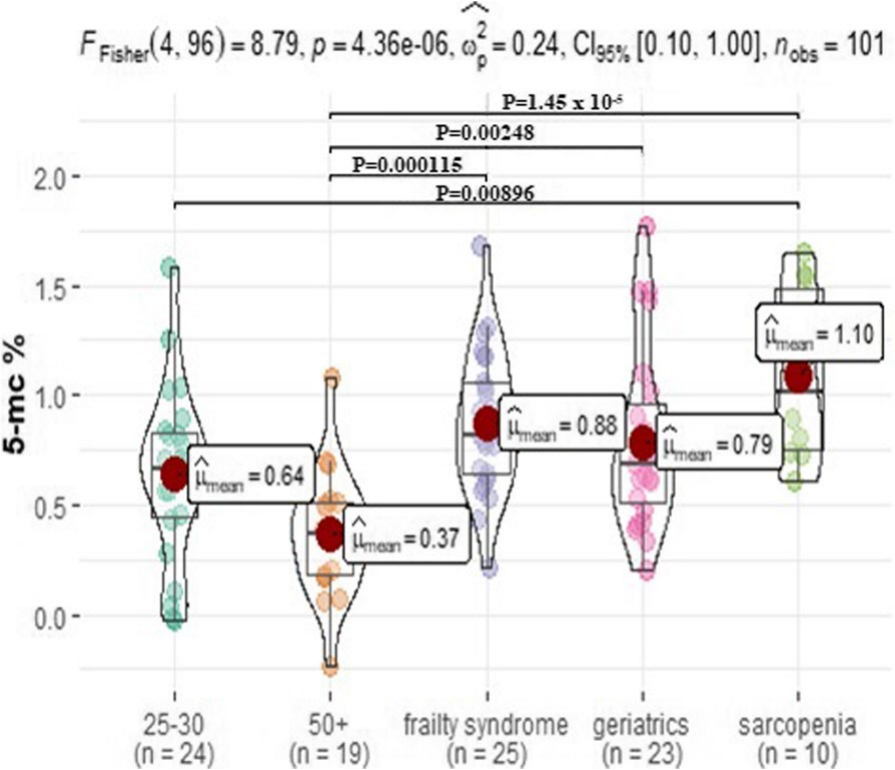
Global DNA methylation in analyzed study groups (healthy subjects at 25–30 age old, healthy subjects at above 50 years old, patients with frailty syndrome, geriatric patients and patients with sarcopenia. The results are shown as the median percentage of methylated DNA (5-mC) of total DNA. *p < 0.05 statistical significance

Results of global methylation level are shown in Fig. 3 and Table 2. In the control healthy subjects above 50 years old methylation level was the lowest whereas in patients with sarcopenia it was the highest. Significantly higher levels of methylation were noted in patients with sarcopenia when compared to the healthy subject aged 25–30 (*p* = 0.01) and the healthy subject above 50 years old (*p* < 0.0001). Patients with frailty syndrome were characterized by significantly higher global methylation levels than healthy subjects above 50 years old (*p* < 0.0001). Additionally, global methylation was statistically higher in geriatric patients than in healthy subjects aged 50 + (*p* = 0.003).

**Table 2:** Global DNA methylation (% of methylated DNA) in analyzed groups

	**Mean (SD)**	**Median (IRQ)**	**Min–max**	**N**
Sarcopenia	1.1 (0.39)	1.02 (0.72)	0.61–1.65	10
Frailty syndrome	0.88 (0.32)	0.82 (0.41)	0.22–1.68	25
Geriatric control	0.79 (0.41)	0.69 (0.45)	0.20–1.77	23
HC 25–30	0.64 (0.39)	0.66 (0.38)	-0.03–1.59	24
HC 50 +	0.37 (0.29)	0.37 (0.33)	-0.23–1.08	19

*HC 25–30* Healthy control subjects at 25–30 years old, *HC 50* + Healthy control subjects above 50 years old, *SD* Standard deviation, *IRQ* Interquartile range, *N* Number of analyzed subjects in the group

**Fig. 4:**
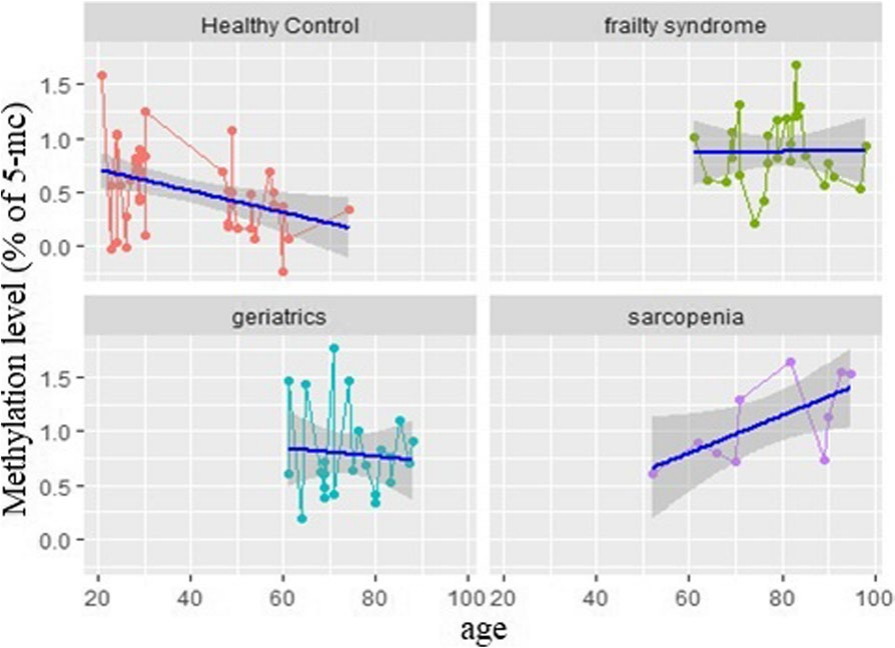
Correlation between methylation level (% of 5-mc) and age in healthy control, patients with frailty syndrome, geriatric control and patients with sarcopenia

Additionally, it has been observed that in healthy subjects methylation decreases with age, but increases in patients with sarcopenia. In the group of patients with frailty syndrome and geriatrics control, the association between age and methylation level has not been observed, as shown in Fig. 4.

### Relative telomere length

**Fig. 5:**
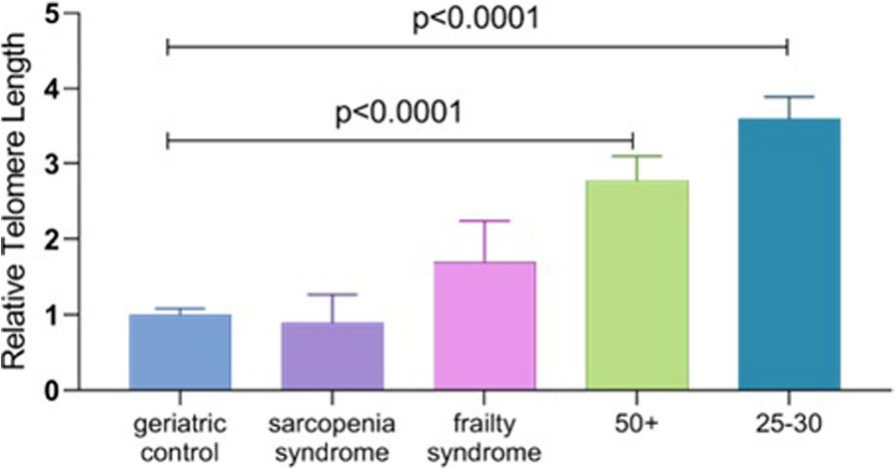
Comparison of relative telomere length between patients with sarcopenia, frailty syndrome, healthy subjects above 50 years old (50 +) and healthy subjects at age 25–30 years old (25–30) in comparison to geriatrics control. Geriatric control was taken as 1. *p < 0.05 as statistically significant

Relative telomere length was analyzed by comparative ∆∆Cq method presented in Fig. 5. The analysis did not show statistical significance in sarcopenia syndrome and frailty syndrome in comparison to any of the studied groups. Significantly longer telomeres were observed in both healthy patients; aged 25–30, and in patients aged 50 + in comparison to geriatric control (*p* < 0.001).

### Total oxidant status

**Fig. 6:**
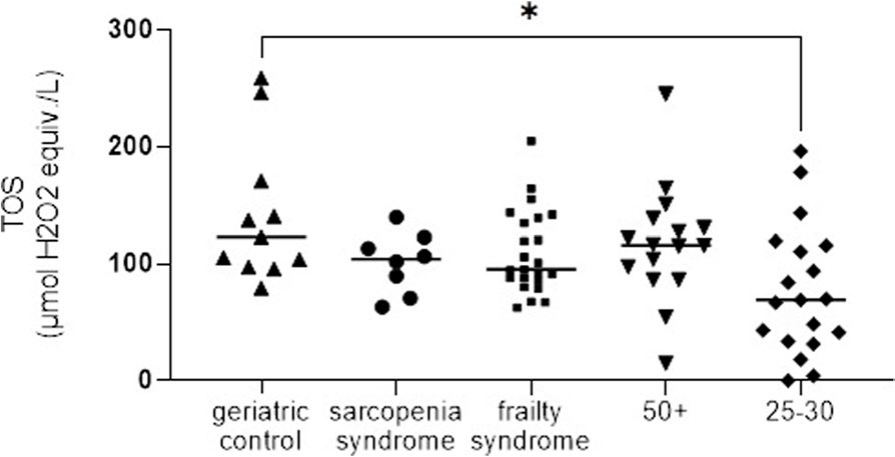
The comparison of total oxidant status between five study groups

The analysis of TOS showed no statistical significance between the groups consisting of patients aged 50 + but it has shown significantly higher TOS values in geriatric control compared to healthy control patients aged 25 – 30 years old (*p* = 0.0232) presented in Fig. 6.

## Discussion

The world population is rapidly aging, which is correlated with the growth of many aging-related diseases such as cancer, heart disease, neurodegenerative diseases, obstructive diseases, and type 2 diabetes, but also with aging-related complications and vulnerability of infectious common diseases. Therefore, there is a need to increase the number of studies and development of therapies against degenerative aging processes [3, 24, 25]. Moreover, a better understanding of the aging process will allow a better understanding of variations in biological aging and age-related diseases.

The goal of this study was to investigate how methylation, telomere length and total oxidant status affect patients with geriatric syndromes. We focus on sarcopenia and frailty syndrome, which were compared to geriatric patients without these entities at average age 77 years (between 52 – 95 years old). Additionally, healthy controls were divided into two subgroups according to their age; 1) young control named 25–30 with a median of 27 (21—30), and 2) elderly control named 50 + with a median of 54,5 (47—74). In our groups of patients, we noticed a correlation between different clinical parameters related to the nutritional status of our patients. In the sarcopenia group, we observed a positive correlation between the level of vitamin D3 and vitamin B12. Symptoms of deficiency of both vitamins result in muscle weakness, weight loss and fatigue which are all hallmarks of sarcopenia. Whereas, in frailty syndrome, we stated a negative correlation between ASMM and level of vitamin D3.

Recently, global DNA Methylation is one of the most often used methods in the analysis of epigenetic factors. This technique relates to referring to the total level of 5mC content in a sample compared to the total cytosine content [18]. In the present study, we found that the global methylation level was the lowest in healthy controls over age 50 (5-mc % mean 0.37) whereas the highest level of global methylation was noted in patients with sarcopenia (5-mc % mean 1.10) healthy control 25–30 (5-mc % mean 0.64), frailty syndrome (5-mc % mean 0.88), and geriatrics control (5-mc % mean 0.79). We were able to observe global methylation levels decreased with age of patients which were in the 50 + group, but statistically significant different level of methylation were presented between sarcopenia patients with healthy control 25–30 (*p* = 0.01) and with healthy control 50 + (*p* < 0.0001), and between patients with frailty syndrome with healthy control 50 + (*p* < 0.0001). However, studied entities and health status have a significant impact on global methylation levels. Interestingly, the present study revealed a significantly higher level of methylation in patients with sarcopenia compared to healthy subjects aged 25–30, and above 50 years old. On the other hand, patients with frailty syndrome and geriatrics patients were characterized by significantly higher levels of methylation only when compared to healthy subjects above 50 years old but not to healthy subjects aged between 25–30 years old. We were unable to find a difference in methylation level between patients with sarcopenia and in frailty syndrome in future studies it would be interesting to see differences in global DNA methylation in larger study groups with age and sex matched controls. This area of research is relatively new and there are a limited number of studies describing the association between methylation and aging. Turner et al. identified epigenetically modified genes, such as *FLNB, MYH9, SRGAP1, SRGN, ZMIZ1* which were associated with gene expression across both the transcriptome and methylome. However, their role in skeletal muscle regulation requires more investigation [26]. Gensous et al. proved that aging is strongly associated with changes in DNA methylation. DNA methylation and epigenetic changes have been directly connected to longevity in many different organisms. The main direction proved by many in vitro and in vivo studies is promotion of global hypomethylation (non-CpG islands) and regions of hypermethylation (mainly CpG islands) with age. A better understanding of the pathways which are involved with muscle aging and sarcopenia is necessary to improve diagnostic methods and treatment of muscle loss with age [27]. Obviously, the functional impact of the age-related methylation changes in sarcopenia and frailty syndrome requires more study. According to our knowledge, this is the first study on global DNA methylation and aging impact on sarcopenia and frailty. Nevertheless, DNA methylation patterns should be evaluated in a larger group.

Age impact has also been confirmed in relative telomere length analysis. However, we did not reveal the potential impact of sarcopenia and frailty syndrome on relative telomere length. The present study showed significantly the longest relative telomere length in a control group aged 25–30, which is in line with the dogma of science. Additionally, analyzed geriatric patients were characterized by lower relative telomere length results in comparison to both healthy subject groups. Based on our results we noticed shorter telomeres length in patients with sarcopenia than in other groups, but the differences were not statistically significant probably because of the group size. We observe shorter telomeres in sarcopenia patients when compared to frailty syndrome but we were unable to find a statistically significant difference. It would be interesting to repeat relative telomere length analyze on larger group of patients with sarcopenia and with frailty syndrome, it might allow to observe statistically difference between both research groups which would be one of molecular mechanism which could be used in future for more accurate diagnosis and treatment sarcopenia and/or frailty syndrome. Bernabeu-Wittel et al. showed that oxidative stress and telomere shortening were higher in blood samples in patients with sarcopenia and/or frailty syndrome compared to patients without these two geriatric syndromes. Telomere shortening was related to decreasing functionality. Moreover, oxidative stress markers with telomere shortening were associated with a higher fatality rate. According to the authors both of these biomarkers can be useful in the clinical evaluation of susceptible patients predisposed for sarcopenia and frailty syndrome [28]. Other researchers proved that telomere length is affected not only by age but also by lifestyle, psychological condition, and disease, such as sarcopenia [29, 30]. In the present study telomere length was shorter in patients with sarcopenia than in patients with frailty syndrome. However, according to Cederholm et. al differences at a molecular level may stem from different catabolism status in muscles observed in sarcopenia (specific muscle loss) and frailty syndrome (weight loss in general) [31].

In 1985, Sies created a concept of oxidative stress which is related to an excess of pro-oxidative factors and reactive oxygen species, over anti-oxidants [32]. An imbalance between these factors can lead to oxidative damage of lipids, proteins, carbohydrates, as well as DNA [33]. The precise mechanism of oxidative stress-inducing aging is still unknown [34]. Nevertheless, it has already been described in age-related diseases, like cardiovascular diseases, diabetes, chronic kidney disease, osteoarthritis, sarcopenia and frailty syndrome [35]. The present analysis of TOS in serum showed statistically significant higher values in geriatric control compared to healthy subjects aged 25–30. All patients with frailty syndrome, sarcopenia and the geriatric control group, as well as healthy subjects over the age of 50, had higher TOS levels than healthy subjects aged 25–30 we observe a similar level of TOS between 50 + with geriatric controls which were the highest from all analyzed groups, after which the level of TOS in sarcopenia and frailty were on very similar levels. Differences between research groups were very slim and larger patients count in each group would not make a difference TOS level which excludes TOS as molecular mechanism which could be used for differentiation sarcopenia from frailty patients. In this analysis, we also observed wide individual variation in the TOS parameter. Apart from different methods of oxidative stress analysis, Bernabeu-Wittel et al. in their study found that patients with sarcopenia and/or frailty syndrome have a significantly higher level of superoxide dismutase (SOD) in plasma in comparison to other poly-pathological patients. Authors found also higher levels of other oxidative stress markers such as CAT (Catalase), GR (Glutathione reductase) in sarcopenia and frailty syndrome, as well as lower levels of TAC-ROS (Total antioxidant capacity reactive oxygen species) in sarcopenia patients, but these differences were not statistically significant [28, 36]. Unfortunately, the present study was limited in several ways. One of them was a sample size, particularly in the case of patients with sarcopenia. In the present study we included patients suffering from sarcopenia or frailty syndrome but not both entities, therefore in further studies it would be recommended to analyze patients with both entities.

In conclusion, the results of our study show that the main factor affecting methylation, telomeres length and level of total oxidant stress is the age of patients. With the number of patients which were qualified for this study was insufficient for accurately analyzing differences between two syndromes, and as a result understanding molecular mechanisms which allow studies to develop syndromes. At this stage of the experiments, we were unable to find markers distinguishing sarcopenia and frailty syndrome. In future experiments the number of patients in each group should be increased.

## Abbreviations

ADL: Activities of daily living
ASMM: Appendicular skeletal muscle mass
BMI: Body mass index
CAT: Catalase
CGE: Comprehensive geriatric evaluation
CRP: C-reactive protein
DEXA: Dual-energy x-ray absorptiometry
DNMT: DNA methyltransferase
FI-CGA: Frailty index-comprehensive geriatric assessment
GR: Glutathione reductase
LDL: Low-density lipoprotein
IADL: Instrumental activities of daily living
POMA: Performance oriented mobility assessment
RTL: Relative telomere length
SCR: Single copy reference
SOD: Superoxide dismutase
TAC-ROS: Total antioxidant capacity reactive oxygen species
TLR: Telomere primer solution
TOS: Total oxidant status
TUG: Time up and go
VES-13: Vulnerable elder scale-13
